# Characteristics of Lower Leg Muscle Activity in Patients with Cerebral Palsy during Cycling on an Ergometer

**DOI:** 10.1155/2018/6460981

**Published:** 2018-05-09

**Authors:** Susmita Roy, Ana Alves-Pinto, Renée Lampe

**Affiliations:** ^1^Research Unit of the Buhl-Strohmaier Foundation for Cerebral Palsy and Paediatric Neuroorthopaedics, Orthopaedic Department, Klinikum rechts der Isar, Technical University of Munich, Munich, Germany; ^2^Markus Würth Professorship, Technical University of Munich, Munich, Germany

## Abstract

**Purpose:**

Cycling on ergometer is often part of rehabilitation programs for patients with cerebral palsy (CP). The present study analyzed activity patterns of individual lower leg muscle during active cycling on ergometer in patients with CP and compared them to similar recordings in healthy participants.

**Methods:**

Electromyographic (EMG) recordings of lower leg muscle activity were collected from 14 adult patients and 10 adult healthy participants. Activity of the following muscles was recorded: Musculus tibialis anterior, Musculus gastrocnemius, Musculus rectus femoris, and Musculus biceps femoris. Besides qualitative analysis also quantitative analysis of individual muscle activity was performed by computing the coefficient of variation of EMG signal amplitude.

**Results:**

More irregular EMG patterns were observed in patients in comparison to healthy participants: agonist-antagonist cocontractions were more frequent, muscle activity measured at specific points of the cycle path was more variable, and dynamic range of muscle activity along the cycle path was narrower in patients. Hypertonicity was also more frequent in patients.

**Conclusion:**

Muscle activity patterns during cycling differed substantially across patients. It showed irregular nature and occasional sharp high peaks. Dynamic range was also narrower than in controls. Observations underline the need for individualized cycling training to optimize rehabilitation effects.

## 1. Introduction

Cerebral palsy (CP) is a symptom complex that is caused by early brain damage [1, 2]. In the foreground there is a restriction in posture or in motor function that affects mobility and often also manual skills. Depending on the extent of brain damage, restrictions in mobility can vary from patients being able to walk without the need of a walking aid to patients being confined to a wheelchair. The most common form of the condition is spastic CP, characterized by elevated muscle tone (i.e., hypertonicity) [3, 4] and increased resistance to movement that affects the smoothness with which these are performed. In addition to this, spastic muscle tone can lead to joint contractures, which together with a limitation of the walking ability can lead to malposition. Nevertheless, also muscle weakness occurs frequently [5].

The loss of the motor control in spastic CP can result in synergistic muscle activity [6]. Also selective motor control, considered as the capacity for voluntary use of individual muscles, is disturbed [7]. In the case of a spastic muscle tone, the measured muscle activity reflects not only contraction resulting from activation, but also activity resulting from passive muscle stretching or contraction against muscle resistance. In fact, in electromyography (EMG) recordings, muscle tension resistance can be more pronounced than development of strength of the muscle itself.

Cycling on a moving or stationary bicycle or on an ergometer is a standard part of rehabilitation programs in CP, as cycling promotes movement and therefore promotes the development of muscle strength of lower leg mobility, especially important for patients that are confined to a wheelchair, and as a therapy to prevent joint contractions and to increase muscle strength. The importance of cycling for training motor function in CP has led to the development for example of adapted bicycles for dynamic cycling [8, 9], and of therapeutic programs involving Functional Electrical Stimulation [10].

Previous studies have reported that during cycling in a stationary bicycle children with CP tend to cycle irregularly [11], in contrast to typically developing peers that tend to cycle at a regular pace. EMG recordings of lower leg muscle activity during cycling have furthermore shown muscle weakness [12] and altered muscle activation patterns in children and adolescents with CP, with earlier onsets and later offsets of activity [13], longer ranges of activation along the cycle path [11, 12], more frequent cocontractions [11–13], and decreased muscular efficiency [13].

Given that cycling is a relevant component in the therapy of patients with CP and given the above-mentioned alterations in muscle activity in CP relative to healthy persons, it would be important to develop training methods that could promote a more physiological pattern of cycling. Training methods that take into account the neuronal origin of the posture and motor deficits in CP are likely be more therapy-effective. These developments require, however, a deeper insight into the neuropathological processes underlying altered EMG activity during cycling.

Previous research employed EMG to describe pathologies in muscle activity in people with congenital and acquired brain damage when compared to healthy controls, especially when performing a task. However, a differentiated characterization of the muscle activity signals produced by a spastic muscle is rarely discussed in EMG studies.

The purpose of the current study was to analyze individual muscle activity during active cycling on an ergometer in persons with CP and compare it to similar recordings in healthy persons. The study here presented extends the findings described in a previous work [14] that focused on the coordination between agonist and antagonist muscles, as well as on the coordination between the two legs. Deviations in EMG activity were furthermore positively correlated with the degree of spasticity and mobility. The work now presented, rather than analyzing coordination between muscles, focusses on the physical characteristics of EMG signals of individual muscles by themselves by looking into EMG signals recorded: (1) at specific positions as well as (2) during the whole cycle path. Quantitative analysis of movement quality in terms of EMG signal amplitudes may be a useful tool in the development of targeted rehabilitation.

## 2. Methods

### 2.1. Participants

Fourteen adults with CP and ten healthy adults participated in this study. Patients with CP were aged between 23 and 60 years. They were recruited from a day care center for persons with disabilities. Healthy participants were aged between 25 and 55 years. They were recruited among the personnel working at the hospital.

The degree of patient mobility was expressed according to the Gross Motor Function Classification System (GMFCS) [15]. This system defines five different levels of mobility: from a GMFCS of I, when the patient is able to walk freely without the need of a walking aid, to a GMFCS level of V when patients have substantial motor limitations and seat permanently in a wheelchair, not being able to move by themselves. GMFCS levels of the participants of this study varied from GMFCS of I to GMFCS of IV (Table 1).

The degree of spasticity of lower legs was characterized according to the Modified Ashworth Spasticity (MAS) scale [16]. This scale ranges from 0 to 4, with level 4 corresponding to the highest degree of spasticity.

### 2.2. Experimental Procedure and Data Analysis

EMG data of lower leg muscle activity were collected during active cycling on an ergometer. Activity was recorded from the following muscles: Musculus tibialis anterior (M. tibialis anterior), Musculus gastrocnemius (M. gastrocnemius), Musculus rectus femoris (M. rectus femoris), and Musculus biceps femoris (M. biceps femoris). Recordings were performed with a self-developed EMG [14] system with 8 channels and surface electrodes. The system includes hardware implementation of the following signal processing steps: amplification, low-pass filtering with a cutoff frequency of 2-Hz, and a final amplification before digitizing the signal with a 77-Hz sampling rate. The experimental procedure has been described elsewhere [14].

All the experimental procedures were approved by the ethics committee of the Faculty of Medicine of the Technical University of Munich before starting data collection. Participation in the study was voluntary, and all subjects, or in some cases their legal protectors, gave their written informed consent before performing the tests.

Evaluations were developed with the aim of finding the typical physiological and pathological patterns in EMG during cycling, and to allow comparison in terms of similarities or differences between healthy and participants with CP.

**Figure 1:**
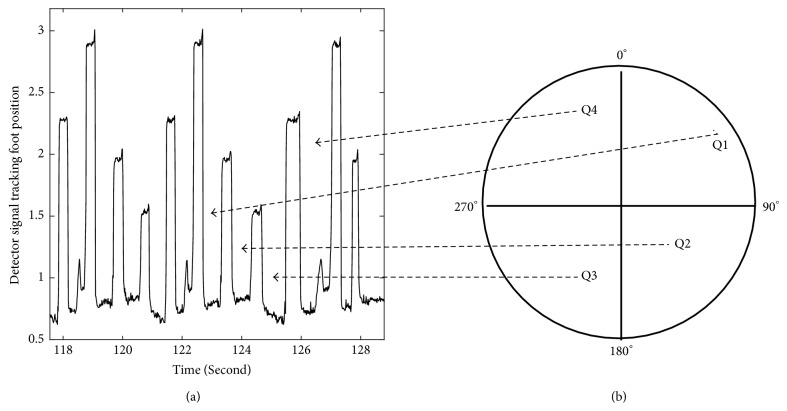
The position of the foot during cycling was tracked with a system of detectors fixed to the wheel of the ergometer. (a) The left panel shows the signal emitted by the detector for two and half revolutions. Each peak in the signal indicates a specific position of the foot during the cycling: for example, the maximum signal amplitude (highest peak in the signal) was emitted when the foot reached at the 0° position. (b) The periods of time between the peaks correspond to the times spent in each of the four quadrants, as indicated.

During cycling the path traveled by the foot/leg is circular, with the position of the foot in any instant being defined by the angle formed by two radial lines: one stretching from the center of the wheel to the highest point possible, the 0° (panel (b) in Figure 1), and the other one stretching from the center of the circle and the point where the foot is located. Hence, the recorded full revolution periods (360° epochs) of muscle activity signals can be analyzed as a function of the wheel/foot position along the full revolution path, namely, as a function of the position defined by the quadrant, as illustrated in Figure 1. The foot itself could not move freely; it was strapped to the foot holder of the bicycle. During EMG recordings, participants were asked to cycle as regularly as possible and the analysis here presented refers to periods of active muscle use.

**Figure 2:**
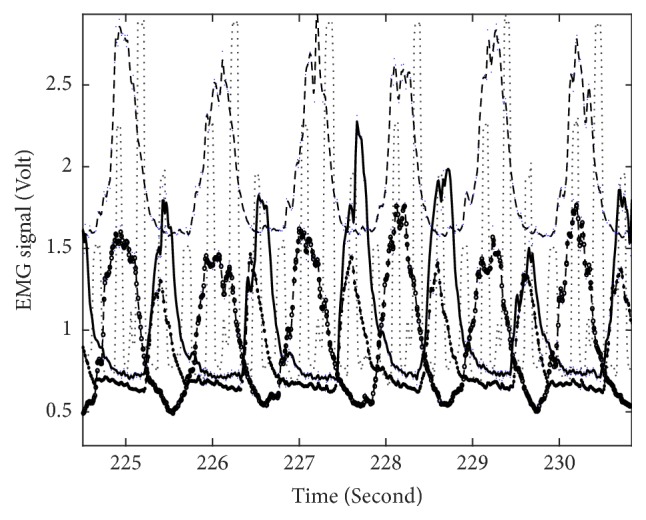
EMG signal recorded from four leg muscles in a healthy participant during circa five cycling revolutions. Solid curve illustrates EMG recorded from M. rectus femoris, the long dashed curve M. biceps femoris, dashed with circle curve M. tibialis anterior, and dash dot line M. gastrocnemius. The foot position during the same cycling interval is indicated by the dotted line.

**Figure 3:**
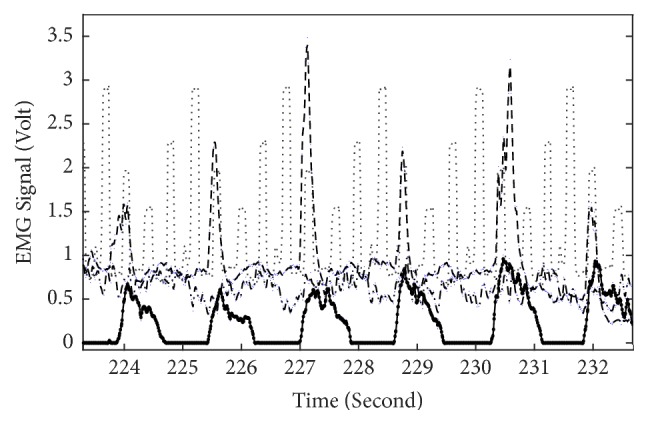
Similar to Figure 2 but for a patient with CP. Line-muscle correspondence as in Figure 2.

Figure 2 illustrates example EMG recordings from the four recorded muscles in one leg as a function of time, for one healthy participant. Besides the activity of each muscle also the foot position is depicted (thin dotted line). Similar data is represented in Figure 3 for a patient with CP.

*Different Types of Analysis Were Performed*

(1) A qualitative analysis identified, for each muscle, the quadrant of maximum EMG amplitude during the full cycling revolution. The patterns of activity between muscles in one leg, between the two legs, and between patients and healthy participants were compared. This analysis for all the twenty-four participants is summarized in Table 2.
(2) For each muscle, EMG amplitudes at specific points of the cycling path, 0°, 90°, 180°, and 270°, were obtained for consecutive cycles and the coefficients of variation (CV) at each of these positions were computed: CV is defined as the ratio of the standard deviation *σ* to the mean *μ*:
  (1) CV=σμ×100%σ=1n−1∑i=1nxi−μ2,  μ=1n∑i=1nxi.

 
*n* is number of data points, *x*_*i*_ is EMG amplitude of sample *i*.
  This computation gives an indication of how regular the EMG activity is every time a given wheel position is reached.

(3) For each muscle, the coefficient of variation along the full cycle (and not at a specific point in the cycle as in (2)) was computed. This gives an indication of the range of EMG amplitude throughout the full cycle.

The results presented below were derived from the analysis of EMG patterns, from all the twenty-four participants. All the analyses were performed with customized code in MATLAB2017a.

## 3. Results

Table 2 indicates the quadrant at which each muscle of the left leg was most active during cycling. It is clear from this table that, for example, for healthy participants the M. rectus femoris shows strongest activity in the 2nd quadrant, while the antagonist muscle, M. biceps femoris shows maximum activity in the 4th quadrant. In addition to this, and as observed in Figure 2, when one of these muscles has maximum activity the other one has a minimum, and hence, they are active in opposite parts of the cycle and there are no cocontractions. Although not shown, all the healthy participants show this feature quite clearly. A similar feature could be observed for the M. tibialis anterior and M. gastrocnemius: maximum activation occurs in opposite parts of the cycle. This is observed for all the healthy participants and in a consistent way throughout the active cycling time. As expected, similar data are observed for the right leg but shifted by ~180° and therefore not shown here.

On the other hand, patients with CP show a much more irregular activity pattern in several respects. Firstly, it is clear from Table 2 that there is no muscle for which maximum activity occurs at the same quadrant for all patients, like in the case of healthy participants. For example, while all healthy participants show maximum M. biceps femoris activity in the 4th quadrant, only three patients with CP show maximum activity in the 4th quadrant, five others in the 3rd, and two in the 1st quadrant.

Secondly, for 57% of the patients M. rectus femoris and M. biceps femoris do not show the opposite activation as observed in healthy participants. That means that for only 43% of the patients the maximal ranges of M. rectus femoris EMG amplitude coincide with minimal amplitude ranges of M. biceps femoris and vice versa. M. tibialis anterior and M. gastrocnemius also do not follow that alternating pattern. Although not shown, ~180° phase shift behaviour of right and left leg is not observed in patients as in healthy participants. In addition to this, they are very irregular in following the same pattern throughout the active cycling period.

Thirdly, very sharp maxima can be observed for some muscles in 36% of the patients (Figure 3). This might reflect hypertonicity [5, 12].

**Figure 4:**
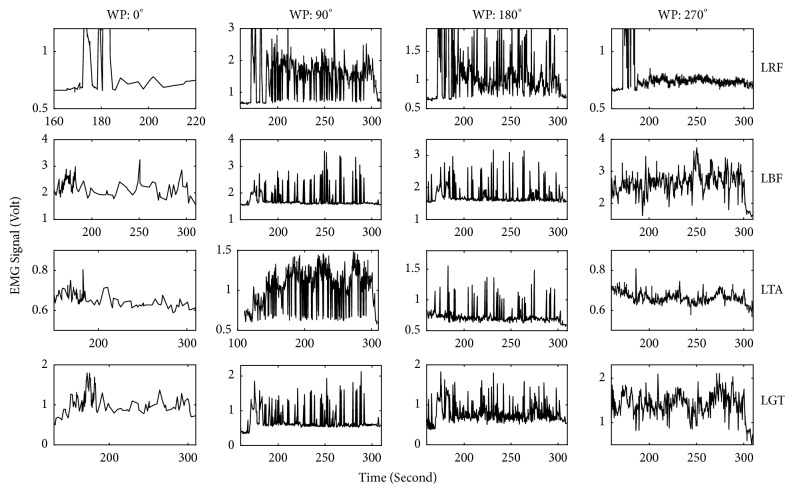
EMG signal recorded from four leg muscles at four wheel positions over the complete active cycling, for a healthy participant. WP denotes the wheel positions of the ergometer. LRF: left M. rectus femoris, LBF: left M. biceps femoris, LTA: left M. tibialis anterior, and LGT: left M. gastrocnemius.

**Figure 5:**
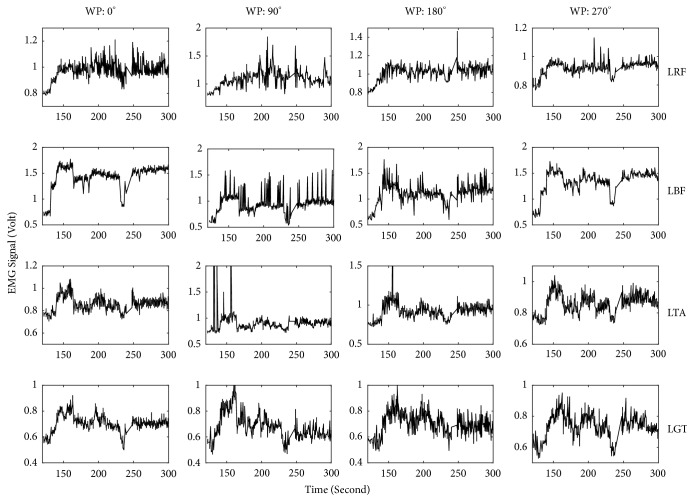
Similar to Figure 4 but for a patient with CP.

**Figure 6:**
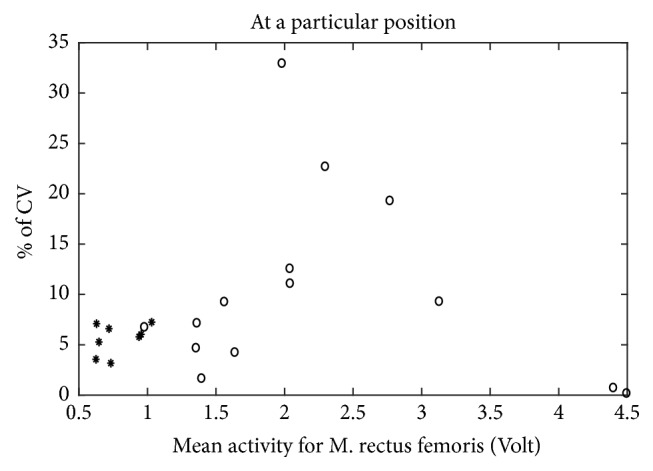
Coefficient of variation of M. rectus femoris at a particular position. *∗*: data for healthy group, ∘: data for patient group.

The amplitudes of the EMG signals recorded at four particular wheel positions during the whole active cycling period were also analyzed. Examples of two such datasets are illustrated in (Figure 4) for a healthy participant, and (Figure 5) for a patient with CP. EMG signals were analyzed for the following four wheel positions: 0°, 90°, 180°, and 270° and the coefficient of variation (CV) was calculated to investigate the extent of variability in relation to the mean of the muscle activation in these samples. This computation was done for all participants and muscles, and the results for the left M. rectus femoris at 90° are plotted in Figure 6. As it is clear from this figure, healthy participants show a CV below 8. This means that the relative variation of muscle activity at this particular position is less than 8%. On the other hand, most patients show CV values above 10%. This means that dispersion of activity around the mean is much larger than for healthy participants.

**Figure 7:**
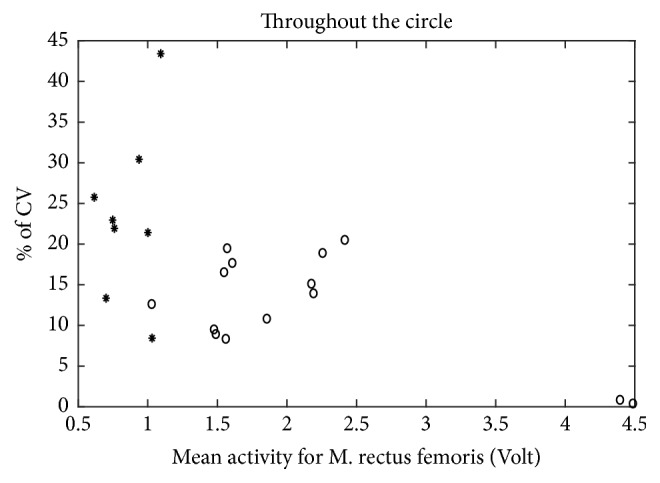
Coefficient of variation for M. rectus femoris over the circle. *∗*: data for healthy group, ∘: data for patient group.

On the other hand, when the CV is calculated over the complete cycle (as presented in Figure 7) for left M. rectus femoris, the result is opposite to that observed in Figure 6: apart from two cases, CV values for healthy participants are larger than for patients (Figure 7), reflecting a larger variation in EMG amplitude throughout a cycle. However, for two healthy participants, the CV lies below 15%, indicating a small variation in M. rectus femoris throughout the circle.

## 4. Discussion

Analysis of the quadrants where each individual muscle is most active and comparison of results between patients with CP and healthy participants (Table 2, Figures 2 and 3) underline the lack of an alternating pattern of activation of opposing muscles (e.g., M. rectus femoris and M. biceps femoris) in patients in comparison to healthy participants reported previously, for example [11, 14]. Also the more frequent occurrence of cocontractions in patients with CP was observed here, together with irregular EMG activity in several muscles (Table 2).

Analysis of EMG recordings at specific positions of the cycling path showed a larger variability in EMG amplitude in the group of patients than in healthy participants (Figures 4, 5, and 6). This means that, in healthy controls, every time the leg reaches a specific wheel position the recorded EMG amplitude is very similar to the previous measurement in that same position. In other words, EMG amplitude at a specific position of the foot is very consistent throughout the cycling. This is not the case for several of the patients, for whom EMG amplitude at that same specific position changed substantially every time the foot returned to that position. This dispersion in muscle activity in the patient group is likely due to the limited sensorimotor control resulting from central brain damage. Associated with this is the observation of a much more sporadic muscular activity and hypertonicity (Figure 3) in the patient group throughout the cycling. This failure in producing a regular and consistent pattern of EMG activity throughout cycling suggests a failure in the mechanisms responsible for activating and regulating muscle activation and, given the origin of cerebral palsy, likely at the level of the central nervous system [17] or neuromuscular mechanisms.

On the other hand, CV values computed throughout a full cycle were lower in patients than in healthy participants (Figure 7). This indicates a less flexible muscle in the case of patients. The less frequent use of muscles in patients can only partly explain this smaller responsiveness of muscles; otherwise intensive training would be enough to change this responsiveness. Another explanation for this lack of modulation may lie in the disturbed reciprocal innervation in CP [3, 5, 18–20]. Also alterations in physical properties of muscle fibers [21] and secondary histological changes in the muscle fibers themselves [19, 22] might have contributed to produce a narrower muscle dynamic range as well as disturbed agonist-antagonist coordination. That is, the deviations in EMG signals during cycling observed might partly result from not only disturbance of central mechanisms of motor control but also deviations in physiological functioning in spastic “muscle-units.” Clearly, the way all these factors determine the degree of spasticity and consequently deviations in EMG activity during active movement (e.g., cycling) is complex and individualized. This means also that rehabilitation strategies need to be adapted individually in order to be effective.

## 5. Conclusions

EMG signals measured from lower leg muscles in patients with CP during cycling on an ergometer were observed to have a narrower dynamic range along the full revolution path, when compared to measurements in a group of healthy persons. Muscle status on successive visits to the same wheel position changed very little in healthy participants but was highly variable in patients. That is, not only muscle coordination but also individual muscle activity, independent of other muscles, is affected in patients with CP during cycling as compared to healthy persons.

## Figures and Tables

**Table 1:** Participants mobility according to GMFCS, and degree of spasticity MAS of the lower limbs.

Patient	GMFCS	MAS	
		L	R
1	I	0	0
2	II	0	1+
3	II	1+	1+
4	III	2	0
5	III	2	2
6	III	0	0
7	III	2	0
8	III	2	2
9	III	1+	1
10	III	2	2
11	III	2	2
12	III	2	2
13	IV	2	2
14	IV	2	3

GMFCS: Gross Motor Function Classification System; MAS: Modified Ashworth Spasticity; L: left; R: right.

**Table 2:** A letter in one cell indicates the quadrant (Q1, Q2, Q3, or Q4 as indicated in Figure 1) at which a given muscle reached maximum activity throughout a full cycle. H indicates an healthy participant, C a patient, *I* very irregular EMG pattern recorded from patients, and *N* poor EMG signal from healthy group.

Muscle	Position			
	Q1	Q2	Q3	Q4
M. biceps femoris	*I I I*		C C C C	C C C H
				H H H H
	C C		C	
				H H H H *N*

M. rectus femoris	C C	C C C C C	C C	C
		H H H H H		
	C C			
		H H H *N N*		

M. tibialis anterior	C	*I I* C C	C *I*	
		H H H H H		
		H H H H *N*		

M. gastrocnemius	C	C H	C C*I I*	C C C C
				H H H H
			C C C C	
				H H H H H
